# Proteomic analysis of rice mutant *pir1* reveals molecular mechanisms triggering PCD and conferring high resistance to bacterial blight

**DOI:** 10.3389/fpls.2025.1652068

**Published:** 2025-08-28

**Authors:** Xinyu Chen, Yujie Zhou, Weifang Liang, Yuhang Zhou, Liujie Xie, Fan Hou, Bingsong Zheng, Jianzhong Li

**Affiliations:** ^1^ College of Plant Protection, Shenyang Agricultural University, Shenyang, China; ^2^ State Key Laboratory for Managing Biotic and Chemical Threats to the Quality and Safety of Agro-Products, Key Laboratory of Biotechnology in Plant Protection of MOA of China and Zhejiang Province, Institute of Virology and Biotechnology, Zhejiang Academy of Agricultural Science, Hangzhou, China; ^3^ Ministry of Agriculture Key Laboratory for Plant Protection and Biotechnology, Institute of Virology and Biotechnology, Zhejiang Academy of Agricultural Sciences, Hangzhou, China; ^4^ Zhejiang Provincial Key Laboratory of Plant Virology, Institute of Virology and Biotechnology, Zhejiang Academy of Agricultural Sciences, Hangzhou, China; ^5^ Zhuji Agricultural Technology Extension Center, Zhuji, China; ^6^ Key Laboratory of Saline-Alkali Vegetation Ecology Restoration, Ministry of Education, College of Life Sciences, Northeast Forestry University, Harbin, China; ^7^ Taizhou Academy of Agricultural Sciences, Taizhou, China; ^8^ Wuwangnong Seed shareholding Co., Ltd., Hangzhou, China; ^9^ National Key Laboratory for Development and Utilization of Forest Food Resources, Zhejiang A&F University, Hang-zhou, China; ^10^ Qujiang District Agricultural Technology Extension Center, Quzhou, China

**Keywords:** disease resistance, *Xoo*, proteomics, ROS, photosynthetic pathways, defense pathways

## Abstract

Most rice mutants exhibit some level of resistance to bacterial blight. This study demonstrates that the rice lesion mimic mutant (LMM) *pir1* possesses enhanced resistance to bacterial leaf blight and triggers the upregulation of multiple pathogenesis-related (PR) proteins. Concurrently, photosynthetic parameter measurements revealed a significant impairment in the photosynthetic electron transport chain and photosynthetic capacity in pir1. Assessments of various stress factors and electron microscopy observations indicated that accumulated reactive oxygen species (ROS) caused severe damage to plant organelles. Utilizing proteomic approaches, we analyzed differentially expressed proteins (DEPs) between *pir1* and its wild-type counterpart. Two-dimensional fluorescence difference gel electrophoresis (2D-DIGE) combined with mass spectrometry (MS) analysis of different leaf positions from both materials identified a total of 321 DEPs, comprising 87 upregulated and 234 downregulated proteins. Bioinformatics analysis of these DEPs revealed their involvement in diverse biological processes, including photosynthesis, carbohydrate metabolism, defense responses, redox homeostasis, and energy metabolism. Analysis of the regulatory network suggests that the mutation *pir1* participates in programmed cell death (PCD), thereby triggering disease resistance responses.

## 1 Introduction

Rice bacterial blight, caused by *Xanthomonas oryzae* pv. *oryzae* (*Xoo*), is one of the three major traditional bacterial diseases of rice in China (Mew, 1987; Shasmita et al., 2023). Epidemiological surveys revealed severe outbreaks of bacterial blight (BB) in areas such as Quzhou and Shaoxing in Zhejiang Province, China, in 2014. These outbreaks resulted in extensive yellowing, chlorosis, and lodging of rice plants, with estimated yield losses exceeding 80%. Since then, both the geographical range and severity of the disease have continued to increase annually (Huadi et al., 2017). Long-term agricultural practice has demonstrated that utilizing resistant cultivars is the most economical, effective, and environmentally friendly strategy for BB control. However, under the long-term selection pressure from the same resistance sources, the pathotypes of *Xoo* readily evolve (Liang et al., 2024). This leads to the emergence and spread of new virulent strains, rendering existing resistance genes ineffective. Consequently, the continuous mining and functional characterization of novel resistance gene resources is imperative.

In nature, plants have evolved sophisticated immune mechanisms to defend against pathogen attacks. PCD is a ubiquitous process in organismal development and serves as a defense mechanism against pathogen infection and abiotic stress by selectively eliminating cells (Dangl et al., 1996; Greenberg, 1997). Most incompatible plant-pathogen interactions trigger an oxidative burst, during which PCD is initiated in infected and adjacent cells to restrict pathogen spread (Apostol et al., 1989; Draper, 1997). Upon sensing pathogen infection, Hypersensitive Response (HR) is rapidly activated at the infection site and surrounding tissues, leading to the sacrifice of plant cells to restrict pathogen invasion (Pankaj et al., 2018; Pitsili et al., 2020).

Rice LMMs are a class of mutants that spontaneously develop necrotic lesions resembling HR symptoms in the absence of pathogen infection; these lesions occur on leaves or leaf sheaths. Studies have shown that most rice LMMs confer durable and broad-spectrum disease resistance through mechanisms involving cell death, ROS accumulation, and activation of defense-related genes (Sha et al., 2023). At present, many rice LMMs have been characterized, with at least 61 causal genes cloned and characterized. Among these, 40 were identified via map-based cloning (Chen et al., 2025; Ruan et al., 2024; Wang et al., 2025). These LMM genes encode diverse proteins with various biological functions and can be classified into functional groups such as transcription and protein translation, the ubiquitin-proteasome pathway, protein phosphorylation, vesicle trafficking, metabolic pathways, and plant hormone signaling. This diversity indicates that multiple pathways are involved in lesion mimic formation. OsNPR1 is a key regulator of Systemic Acquired Resistance (SAR) and confers durable, broad-spectrum resistance in rice. *OsNPR1*-OE plants develop lesion mimic spots on leaves and exhibit enhanced resistance to BB (Chern et al., 2005). The rice *spl33* gene encodes a eukaryotic translation elongation factor 1 alpha protein, comprising a non-functional zinc finger domain and three functional EF-Tu domains. Research suggests that ROS accumulation may cause cell death in the *spl33* mutant, and the activation of multiple defense-related genes, such as *PR1a* and *PBZ1*, likely contributes to its enhanced disease resistance (Wang et al., 2017). The rice *OsRLR1* gene encodes a CC-NB-LRR protein showing high homology to *RPM1* in Arabidopsis thaliana, a canonical NLR gene in the disease resistance pathway. In *OsRLR1*-OE plants, the expression levels of *OsNPR1*, *OsPR1a*, and *OsPR10* is significantly upregulated. This indicates that *OsRLR1* regulates *OsNPR1* expression in rice, thereby activating *OsNPR1*-mediated defense responses. Furthermore, *OsRLR1* interacts with the transcription factor *OsWRKY19* via its CC domain, suggesting *OsWRKY19* may participate in the *OsRLR1*-induced disease resistance response (Du et al., 2021). Other LMM genes participate in the ubiquitin-proteasome pathway (Ren et al., 2023). For instance, Protein SPL11 possesses E3 ubiquitin ligase activity and acts as a negative regulator of plant cell death and defense responses. *SDS2* encodes an S-domain receptor-like kinase, interacts with *SPL11* and causes its phosphorylation. Subsequently, SPL11 ubiquitinates SDS2 to regulate its stability. Mutation of SDS2 partially suppresses the LM phenotype and disease resistance in the *spl11* mutant. In addition, SDS2 interacts with two positive regulatory factors of rice immunity, OsRLCK118 and OsRLCK176. Subsequently, OsRLCK118 phosphorylates the NADPH oxidase OsRbohB, inducing ROS outbreaks in plants during pathogen infection, resulting in a disease-resistant response (Fan et al., 2018). In the *ebr1* mutant, accumulation of *OsBAG4* triggers PCD, while reducing *OsBAG4* expression leads to the inhibition of cell death and disease resistance. *EBR1* encodes an E3 ubiquitin ligase and serves as a negative regulator of PCD and immunity in rice. EBR1 interacts with OsBAG4 and targets it for ubiquitination and degradation (You et al., 2016).

Despite significant progress in identifying rice LMM genes and characterizing some related pathways, substantial gaps remain. Firstly, while transcriptomic studies of mutants exist, comprehensive proteomic analyses of LMMs, particularly those exhibiting strong disease resistance, are lacking. Proteomics is essential for revealing post-transcriptional regulation, protein modifications, and functional protein networks directly driving PCD and resistance. Furthermore, the connection between proteomic changes in LMMs and physiological alterations in stress markers, including antioxidant enzymes and ROS levels, has not been fully explored. Understanding the protein network after transcriptional regulation in LMM is crucial for clarifying the functional mechanism of disease resistance. On the other hand, the diversity of pathogen races and the highly mutable nature of pathogens themselves lead to the rapid breakdown of resistance in bred varieties by newly emerging races, resulting in resistance gene failure.

Therefore, to address these gaps and expedite the cloning of the *PIR1* gene, we conducted an integrated study on the mutant *pir1*, which exhibits high resistance to *Xoo*. This mutant displays not only dwarfism, reduced fresh and dry weight, and pollen sterility, but also develops distinct reddish-brown lesions. This indicates that the function of the target *PIR1* gene may be pleiotropic, as its deletion leads to alterations in multiple phenotypic traits. Therefore, studying the mutant *pir1* is significant for elucidating lesion mimic genes and the interdependent regulatory mechanisms between closely associated functional genes. Previous transcriptomic analyses revealed that lignin biosynthesis and plant hormones are involved in triggering PCD (Chen et al., 2020). Here, we performed comparative proteomic analysis between the mutant *pir1* and its wild type to identify core proteins associated with PCD and disease immunity. Additionally, by measuring key stress physiological indicators, including superoxide dismutase (SOD), catalase (CAT), peroxidase (POD) activities, and hydrogen peroxide (H_2_O_2_) levels, we linked the proteomic changes to functional physiological responses. This work provides a proteomic network of the mutant *pir1*, integrated with physiological data, offering novel insights into the molecular mechanisms behind its robust disease resistance. Simultaneously, leveraging proteomics can significantly accelerate the cloning process of the *pir1* LMM gene.

## 2 Materials and methods

### 2.1 Plant materials

Mutant *pir1* is a lesion mimic mutant from an ethyl methyl sulfone (EMS) mutant library that is derived from the ZJ22 (*Oryza sativa* ssp. *Japonica* cultivar ZJ22). The rice materials and cultivation management methods for proteomics are de-scribed by Chen et al (Chen et al., 2020).

### 2.2 Pathogen inoculation and evaluation

*Xoo* Philippine race 6 (P6, strain PXO99A) was used for pathogen inoculation. *Xoo* was subcultured at 28 °C on potato semisynthetic agar (PSA) medium (potato, 300 g/L; Ca(NO_3_)_2_•4H_2_O, 0.5 g/L; Na_2_HPO_4_•12H_2_O, 2.0 g/L; sugar, 15 g/L; agar, 15 g/L) for 48 h. Inoculates were prepared by suspending bacterial cells in sterile water and adjusting the concentration to OD600 = 0.5-0.8. Immerse the scissors in the bacterial suspension, and the leaves were infected with P6 by using scissors dipped in bacterial suspensions to clip leaves 1-2 cm down from the tip of the leaf blade at the heading stage. A total of at least 30 leaves of each of 10 *pir1* and wild-type rice plants were inoculated with *Xoo*. Approximately three weeks after inoculation, the length of the lesion was measured from the cut surface at the tip to the distal-most position on the leaf to assess the resistance. To quantify bacterial populations, three inoculated leaves per plant were homogenized and resuspended in 10 ml of sterile H_2_O, with bacteria harvested individually. Diluted suspensions were plated on PSA medium, and bacterial growth was determined via colony counting of colony-forming units (CFUs).

### 2.3 Extraction and purification of total protein from rice leaves

Three biological replicates were set up in the experiment, and 2.5 g of leaves were taken from each replicate. The flag leaf was named leaf a, he 2nd leaf (from the top) was named leaf b, and the 3rd leaf (from the top) was named leaf c. The total protein of rice leaves was extracted by the previously described phenol-methanol method (Deng et al., 2007). Protein purification was performed using the 2-D clean up Kit (GE Healthcare, UK). According to the manufacturer’s instructions, the protein concentration was measured using the 2d quantitative kit (GE Healthcare, UK). The absorbance of the sample and the standard solution at 480 nm was measured using a spectrophotometer (Ultrospec1100 pro UV), and the concentration of the tested protein was calculated based on the standard curve.

### 2.4 Fluorescent labeling and 2D-DIGE

The fluorescent dye was equilibrated at room temperature for 5 minutes. Then, 25 μL of anhydrous N,N-dimethylformamide (DMF; moisture content <0.005%, purity >99.8%) was added. The mixture was vortexed for 30 sec until complete dissolution of the dye, followed by brief centrifugation at 1200 g. After centrifugation, a working solution was prepared by mixing the fluorescent dye stock solution and DMF at a 1:1.5 ratio. This mixture was vortexed for 30s and centrifuged briefly to yield the fluorescent dye working solution. Separately, the protein solution pH was adjusted to 8.5 using 100 mM NaOH and/or 1M HCl. 20 μL aliquot of the protein solution was transferred to a new PCR tube, and its concentration was adjusted to 5μg/μL. Subsequently, 10 μL of the protein sample was labeled by adding 1 μL of Cy3/Cy5 fluorescent dye working solution and incubating on ice for 30 min. The reaction was quenched with 1 μL of 10 mM lysine, followed by brief vortexing, centrifugation (1 min), and 10 min incubation on ice. Finally, 3 μL of the Cy2 fluorescent dye working solution was added to the mixture, followed by brief vortexing, centrifugation 30 sec, and incubation on ice for 30 minutes. Labeling was terminated by adding 3 μL of 10 mM lysine, vortexing briefly, centrifuging 1 min, and incubating on ice for 10 min. The labeled sample was divided equally into three aliquots for immediate use in subsequent experiments. 2D-DIGE was done according to the method of Dong et al (Dong et al., 2017).

### 2.5 Gel scanning and image analysis

Scanning was performed using a Typhoon 8600 scanner (Ettan DIGE fluorescence scanner). Following initial testing, regions containing high-abundance proteins were selectively scanned, followed by scanning of the entire gel surface. Exposure levels were set, with pixel resolution selected between 30,000 and 55,000 dpi, and the scan resolution was set to 100 μm. Scanner settings were as follows: Cy3 dye: Resulting images appear blue. Emission filter wavelength: 595/25 nm; Excitation filter wavelength: 540/25 nm. Cy5 dye: Resulting images appear red. Emission filter wavelength: 680/30 nm; Excitation filter wavelength: 635/30 nm. Cy2 dye: Resulting images appear green. Emission filter wavelength: 530/40 nm; Excitation filter wavelength: 480/30 nm.

The scanned 2D-DIGE images were analyzed using DeCyder 2D software (version 7.0). Differential In-Gel Analysis (DIA) and Biological Variation Analysis (BVA) modules were employed for the analysis. Protein spots exhibiting differential expression were identified using a threshold of ≥1.5-fold change in abundance value and a statistically significant difference (p < 0.05) as determined by Student’s t-test.

### 2.6 MS identification of DEPs

Protein samples (500 μg) were mixed with an equivalent volume of 2× rehydration buffer and 2 μL of 1% bromophenol blue. The volume was adjusted to 150 μL using 1× rehydration buffer. Following electrophoresis, the gel was transferred into fixing solution and incubated with gentle agitation on an orbital shaker for 1 hour. After fixation, the fixing solution was discarded and replaced with Coomassie Brilliant Blue working solution. The gel was stained on the orbital shaker for 12 hours. Upon completion of staining, the dye solution was discarded and the gel was rinsed with deionized water (ddH_2_O). The gel image was subsequently captured using a UMAX Power Look 2100XL scanner at a scanning resolution of 300 dpi. MS identification was performed by Applied Protein Technology (APT, Shanghai) and Beijing Genomics institution (BGI, Beijing).

### 2.7 Quantification of gene expression by quantitative real-time PCR

Total RNA was extracted from different leaf positions using Trizol. Genomic DNA was removed by treatment with RNase-free DNase. RNA was reverse transcribed into cDNA using the Hifair^®^ III 1st Strand cDNA Synthesis Kit. qRT-PCR was performed using the 2×Super EvaGreen^®^ Master Mix for HRM under the following conditions: 45 cycles of 95°C for 30 s, 60°C for 45 s, and 72°C for 30 s. Actin was used as the internal control. Three replicates were performed per sample. The relative expression levels of the selected DEPs normalized to the expression level of the internal reference control were calculated using the 2^-△△Ct^ method. The primers were designed with Primer Premier Software and are listed in **Supplementary Table S1**.

### 2.8 Determination of stress factors

Nine stress-related indicators - SOD, CAT, POD, Soluble sugar, Soluble protein, H_2_O_2_, O_2_·^−^, Malondialdehyde (MDA), and Proline - were determined by Wuhan ProNets Testing Technology Co., Ltd, WuHan, China.

### 2.9 Determination of photosynthetic parameters

Measurements were conducted during the rice booting stage, with six biological replicates per treatment group. Leaf physiological indices were measured under clear and windless weather conditions. Soil and Plant Analyzer Development (SPAD) values were measured daily at 12:00 using a CL-01 chlorophyll meter on the flag leaf. The middle region of the leaf blade was selected as the measurement site to record the relative chlorophyll content. Photosynthetic performance was measured using the LI-6400XT portable photosynthesis system (LI-COR Biosciences, USA). Measurements were taken on the middle region of the flag leaf (avoiding the midrib by one-third) for the following parameters: Photosynthetic rate (Pn), Stomatal conductance (Gs), and Intercellular CO_2_ concentration (Ci). All data were collected continuously for three days, and the daily mean values were used for statistical analysis.

### 2.10 Transmission electron microscope

Rice leaf segments from ZJ22 and *pir1* plants were cut to dimensions of 1 mm × 3 mm. Samples were fixed overnight at 4°C in 2.5% glutaraldehyde, followed by rinsing with phosphate-buffered saline (PBS; 0.1 M, pH 7.0). Subsequently, samples were fixed in 0.1 M osmium tetroxide solution for 2 hours and rinsed again with PBS. Dehydration was performed using a graded ethanol series (50%, 70%, 80%, 90%, and 95% ethanol), followed by multiple rinses in absolute ethanol (2–3 times) and multiple rinses in absolute acetone (2–3 times). Subsequently, the embedding agent was permeated. Acetone was mixed with the embedding agent in a 1:1 solution and permeated for 1 hour. Prepare a solution of acetone and embedding agent at a ratio of 1:3 and permeate it for 3 hours. The sample blotting solution was placed into a new tube and penetrated with pure embedding agent overnight. Samples were embedded in pure embedding medium and polymerized at 70°C for 48 hours. The embedded blocks were sectioned, and ultrathin sections were stained with uranyl acetate and lead citrate prior to observation using the TEM.

## 3 Results

### 3.1 Resistance analysis of mutant *pir1*

**Figure 1:**
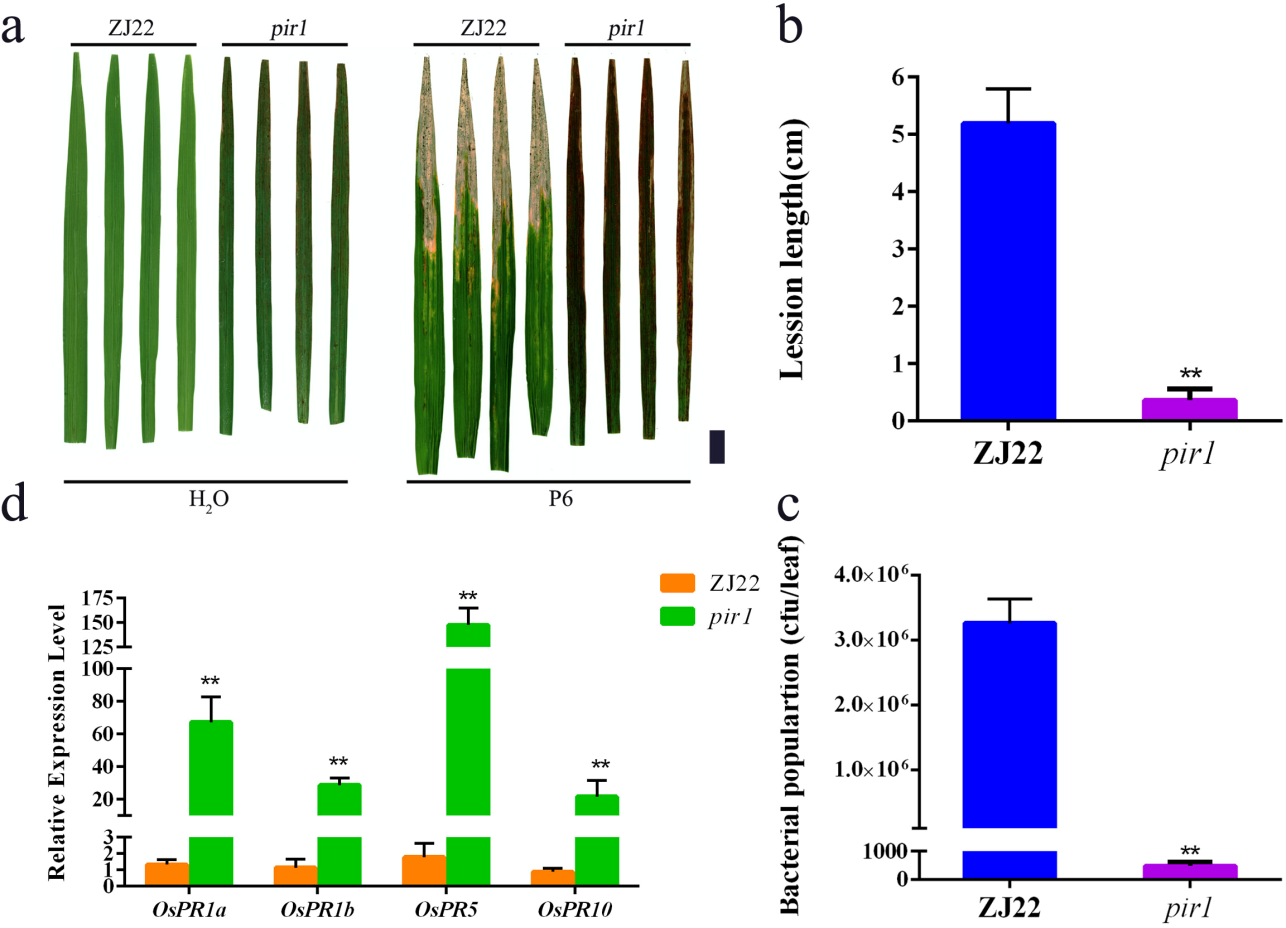
High resistance of *pir1*. **(a)** Resistance identification of wild type (ZJ22) and mutant (*pir1*). Left: Inoculation with water, and right: Inoculation with *Xoo*, Black scale bar = 2 cm; **(b)** Lesion lengths caused by *Xoo* infection. Data are Mean ± SD (n = 15); **P < 0.01; **(c)** *Xoo* population in inoculated leaves at 21 days after inoculation (n = 3*5); **(d)** Relative expression of PRs in ZJ22 and *pir1*. Data are Mean ± SE (n = 3). **P < 0.01.

In this study, *pir1* and wild-type plants were inoculated with P6. Studies have shown that the lesion length of *pir1* was significantly shorter than that of the wild type, and the bacterial count was significantly lower than that of the wild type (**Figures 1a–c**), indicating that the resistance of this mutant to white leaf blight is significantly increased compared with its wild type. The four PRs detected by qRT-PCR, including *OsPR1a*, *OsPR1b*, *OsPR5*, and *OsPR10*, were significantly upregulated in *pir1* compared with wild-type plants (**Figure 1d**). This indicates that *pir1* has typical autoimmune characteristics.

### 3.2 Proteomic analysis

Since the resistance of *pir1* to *Xoo* is closely related to its degree of lesion mimic, we compared the protein profiles of *pir1* in three different leaf positions, as described by chen et al (Chen et al., 2020), Leaf a (a large numbere of lesion mimic spots), leaf b (a small numbere of lesion mimic spots), leaf c (no lesion mimic spots), The leaves at the corresponding positions on wild-type plants were used as controls. 2D-DIGE analysis was used to separate DEPs, and two-dimensional fluorescence electrophoresis images with good repeatability were obtained after scanning (**Supplementary Figure S1**). The 2D-DIGe images of total leaf proteins from three leaf sites of mutant *pir1* and wild-type ZJ22 were analyzed respectively by DeCyder 2D 7.0 software. Protein points with both up-regulation and down-regulation multifactorial of protein expression richness values greater than 1.5 times and p < 0.05 after Student’s t-test were selected. Finally, 325 protein points with significant expression differences were obtained.

**Figure 2:**
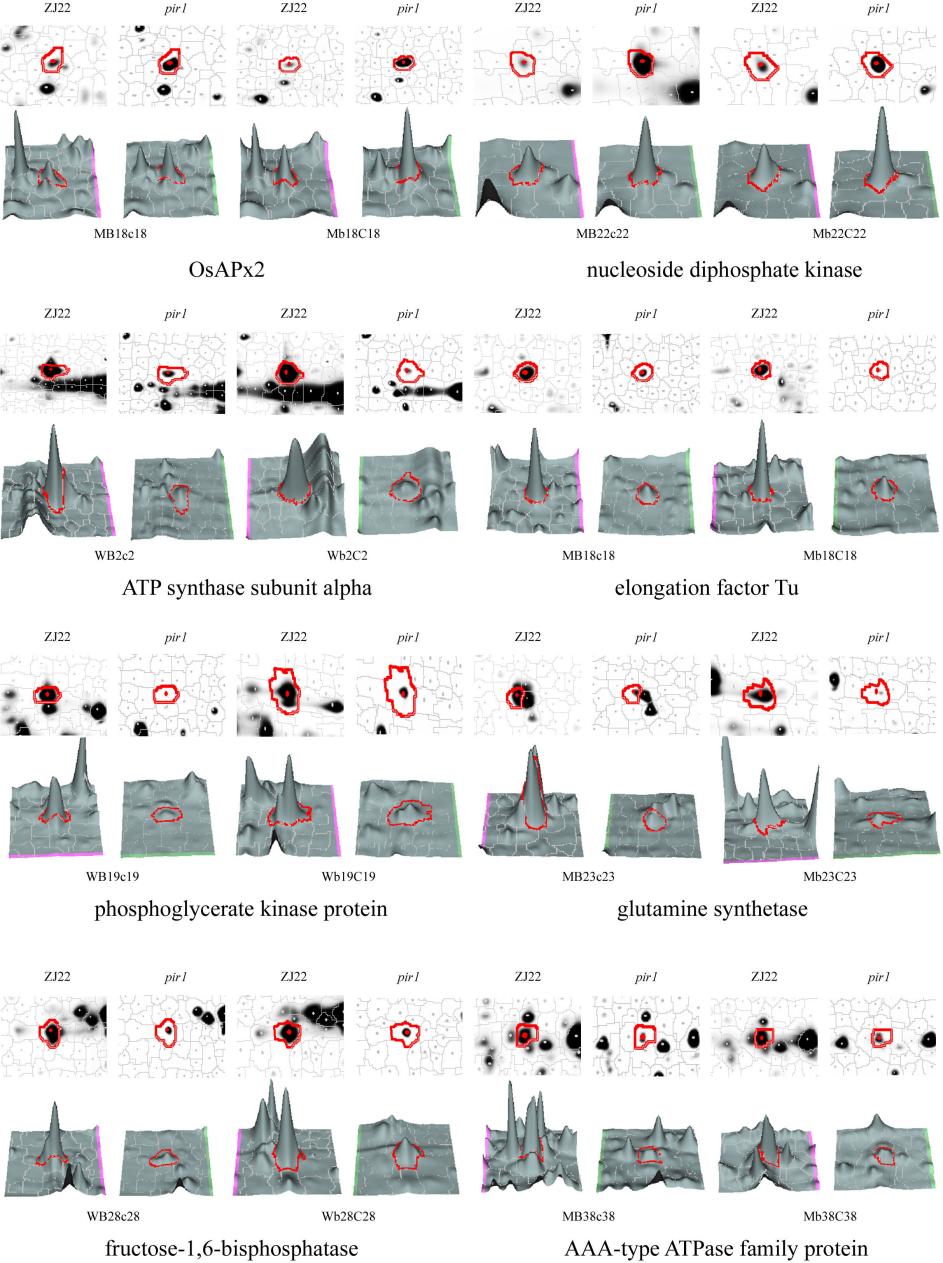
3D view of the expression abundance of some proteins. W represents down-regulated protein spots in *pir1*, and M represents up-regulated protein spots in *pir1*. Capital letter indicates protein spot identified in the corresponding leaf position, for example, MB18c18 indicates that the protein is up-regulated in the b leaf position of *pir1*, and Mb18C18 indicates that the protein is up regulated in the c-leaf position of *pir1*.

After comparing the Coomas brilliant blue staining gel images with the 2D-DIGE images, the differential protein spots were extracted from the Coomas staining gel, and finally 321 protein spots (**Supplementary Table S2**) were identified by MS. Among them, there were 87 protein spots (denoted as M) that were significantly upregulated by the mutant *pir1* compared to the wild type. There were 234 significantly downregulated protein spots (denoted as W) (**Supplementary Table S2**). The three-dimensional stereoscopic images of the expression abundances of some protein spots of ZJ22 and *pir1* identified are shown in the **Figure 2**.

Among these, leaf a exhibited 20 differentially expressed protein sites, while leaf b and leaf c showed 255 and 205 sites, respectively (**Supplementary Figure S2**). This disparity may arise because the mutant’s first true leaf had not yet developed lesion mimic phenotype, maintaining a morphology and physiological profile similar to the wild-type counterpart. Consequently, the differential protein expression at this stage was minimal, accounting for only 9.3% of the total quantified proteins. In contrast, The 2nd and 3rd leaves—stages at which lesion mimic phenotypes emerge—displayed the highest differential expression, representing 79.4% and 63.8% of total proteins, respectively. Notably, 139 proteins were exclusively differentially expressed in both the second and third true leaves but absent in the first true leaf. These proteins likely represent key regulators through which *pir1* triggers PCD and induces disease resistance.

### 3.3 Function classification of DEPs

**Figure 3:**
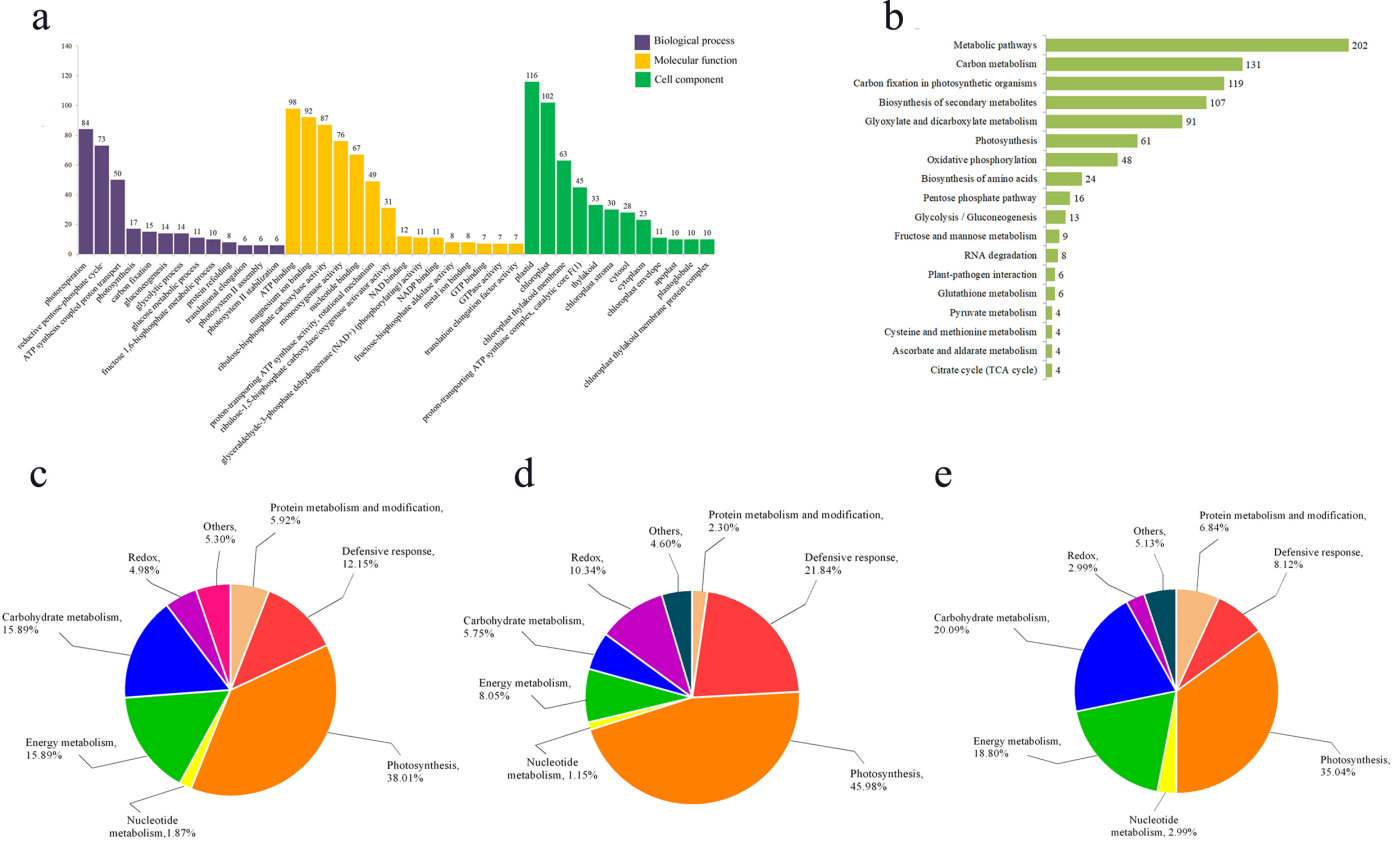
Function enrichment analysis of differentially expressed proteins. **(a)** GO enrichment analysis of differential proteins; **(b)** KEGG enrichment analysis of differential proteins; **(c)** functional analysis of all differentially expressed proteins; **(d)** functional analysis of up-regulated differential proteins; **(e)** functional analysis of down-regulated differential proteins.

We conducted GO enrichment and KEGG pathway enrichment analyses on DEPs respectively. GO enrichment analysis revealed that within Biological Processes, the terms “photorespiration” (GO: 0009853), “reductive pentose phosphate cycle” (GO: 0019253), and “proton transport coupled ATP synthesis” (GO: 0015986) were the most significantly enriched, encompassing 84, 73, and 50 proteins, respectively (**Figure 3a**). In the Molecular Function analysis, “ATP binding” (GO: 0005524), “magnesium ion binding” (GO: 0000287), and “ribulose-bisphosphate carboxylase activity” (GO: 0016984) were the most enriched GO terms, followed by “monooxygenase activity” (GO: 0004497) and “nucleotide binding” (GO: 0000166) (**Figure 3a**). For Cellular Component, the majority of proteins were localized to terms including “plastid” (GO: 0009536), “chloroplast” (GO: 0009507), and “chloroplast thylakoid membrane” (GO: 0009535) (**Figure 3a**).

KEGG enrichment analysis revealed that the DEPs were primarily enriched in the following pathways: metabolic pathways (KO01100), carbon metabolism (KO01200), carbon fixation in photosynthetic organisms (KO00710), biosynthesis of secondary metabolites (KO01110), and glyoxylate and dicarboxylate metabolism (KO00630) (**Figure 3b**).

The functional analysis of the DEPs by using bioinformatics databases such as NCBI, Uniprot, and RGAP, revealed their distribution across various biological processes, including photosynthesis, energy metabolism, defense response, sugar metabolism, and other functions (**Figures 3c–e**). The functional analysis indicated that the largest proportion of DEPs was associated with photosynthesis (38.01%). This was followed by proteins involved in carbohydrate metabolism and energy metabolism (15.89%), while redox-related proteins constituted the smallest group (4.98%) (**Figure 3c**). Furthermore, separate functional analyses of upregulated and downregulated proteins showed that both categories were predominantly enriched in the photosynthetic pathway, accounting for 45.98% and 35.04% of their respective groups (**Figures 3d, e**). This demonstrates that photosynthesis is strongly impaired in the mutant *pir1*. Additionally, defense response-related proteins constituted a significant proportion (21.84%) of the upregulated proteins (**Figure 3d**), suggesting that disease resistance and defense responses are activated in the mutant *pir1*. Conversely, among all downregulated proteins, those associated with carbohydrate metabolism and energy metabolism were relatively abundant, comprising 20.09% and 18.80%, respectively (**Figure 3e**). This pattern is consistent with the observed agronomic traits and characteristics such as the poor fertility in the mutant *pir1*.

### 3.4 Correlation analysis between mRNA and protein expression by qRT-PCR

**Figure 4:**
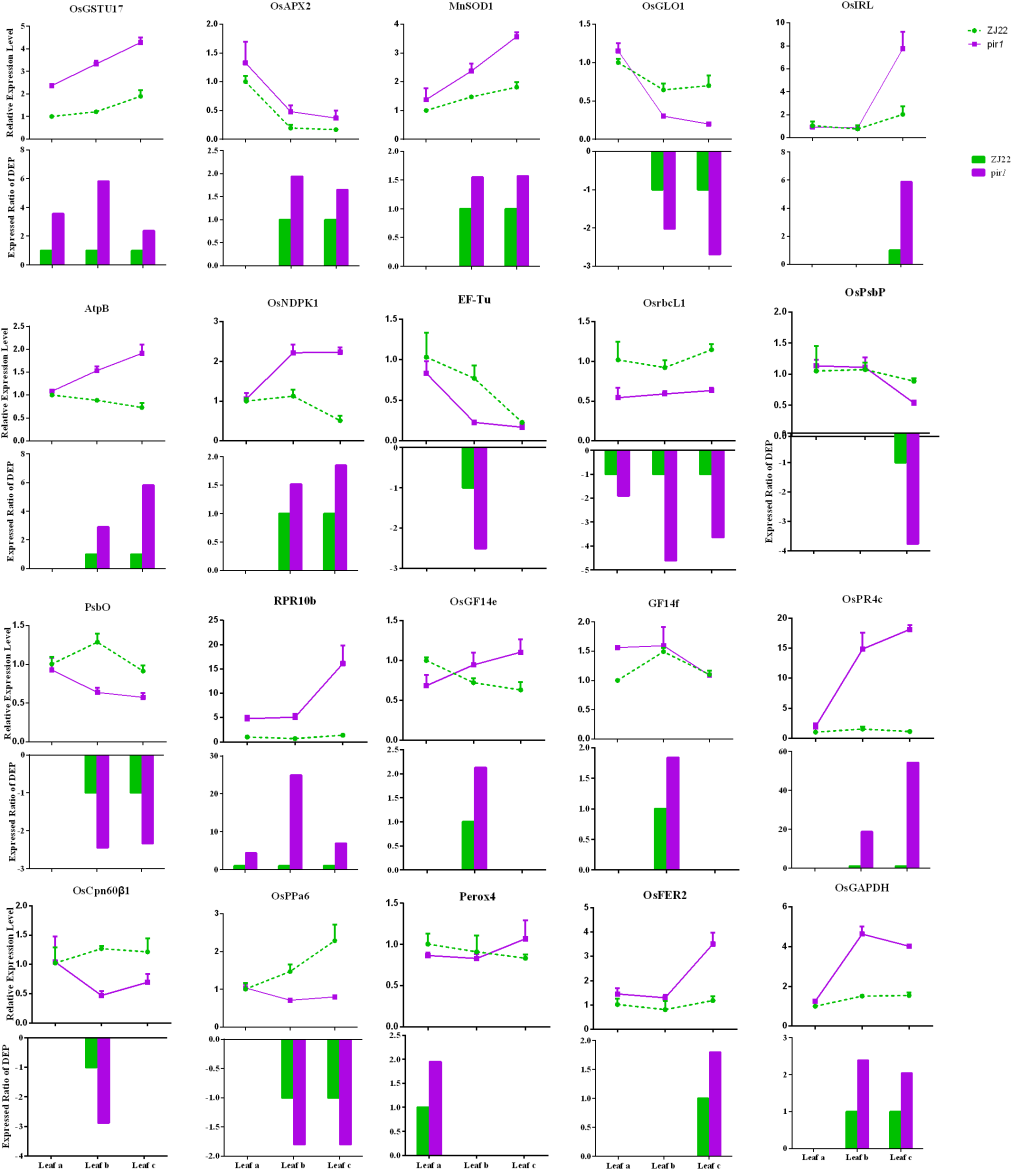
qRT-PCR analysis of differentially expressed genes in wild-type and mutant leaves. qRT-PCR was performed to determine the relative mRNA levels of selected differentially expressed genes (DEGs) in wild-type and mutant leaves. Data represent the mean relative expression level ± standard error (n = 3 biological replicates). In the bar chart, the green dotted line indicates the relative expression level for the wild-type control, while the purple solid line represents the relative expression level observed in the mutant. The bars depict the expression ratio of the mutant relative to the wild type for each DEP.

Changes in DEPs were analyzed at the mRNA level using qRT-PCR (**Figure 4**). **Figure 4** provides a visual overview of the correlation between mRNA and protein levels. To select genes for qRT-PCR validation, particular attention was paid to DEPs identified in key pathways highlighted in this study, such as Redox, Carbohydrate metabolism, Energy metabolism, Photosynthesis, and Defensive response. Furthermore, we noted that these DEPs were also differentially expressed at the transcriptional level in our prior transcriptome comparison of the mutant *pir1* versus wild-type (Chen et al., 2020), indicating strong consistency in the expression of these genes at both transcriptional and post-translational levels (**Supplementary Table S3**). This correlation analysis approach allowed us to investigate the concordance between transcriptomic and proteomic changes in *pir1*. Total mRNA was isolated from leaf a, leaf b, and leaf c of both ZJ22 and *pir1*. The results demonstrate that the vast majority of DEPs exhibit a strong correlation with their corresponding mRNA levels. Specifically, the mRNA levels of the following proteins were highly correlated with changes in their protein abundance: Glutathione S-transferase (MA1B1C1), Ascorbate peroxidase (MB18C18), Manganese superoxide dismutase (MB20C20), Glycolate oxidase (WB86C86), Isoflavone reductase (MC1), ATP synthase subunit beta (MB9C9), Nucleoside diphosphate kinase (MB22C22), Translation elongation factor (WB43), Ribulose-1,5-bisphosphate carboxylase/oxygenase large subunit (Rubisco LSU, WA1B1C1), Photosystem II oxygen-evolving complex protein (WB45), Oxygen-evolving enhancer protein (WB39C39), PR protein (MA2B2C2), 14-3-3-like protein (MB11), PR protein (MB17C17), Chloroplast chaperonin (WB3), Inorganic pyrophosphatase (WB111C111), Ferritin (MC24), and Glyceraldehyde-3-phosphate dehydrogenase (MB1C1). In contrast, 14-3-3-like protein (MB33) and Peroxidase (MA1) exhibited a weak correlation with their respective protein levels in *pir1*.

### 3.5 Analysis of stress factors

Peroxidases and other enzymes involved in high metabolic turnover and self-replication can generate ROS as byproducts of fatty acid β-oxidation. ROS function not only as antimicrobial agents but also as signaling molecules regulating disease resistance responses. In our study, we observed significant enrichment of redox-related proteins. We therefore speculated that the levels of key antioxidant enzymes, such as SOD, ascorbate peroxidase (APX), and POD, might be substantially altered in the mutant *pir1*, leading to ROS accumulation. Consequently, we quantified the levels of relevant stress markers in both WT and mutant *pir1*.

**Figure 5:**
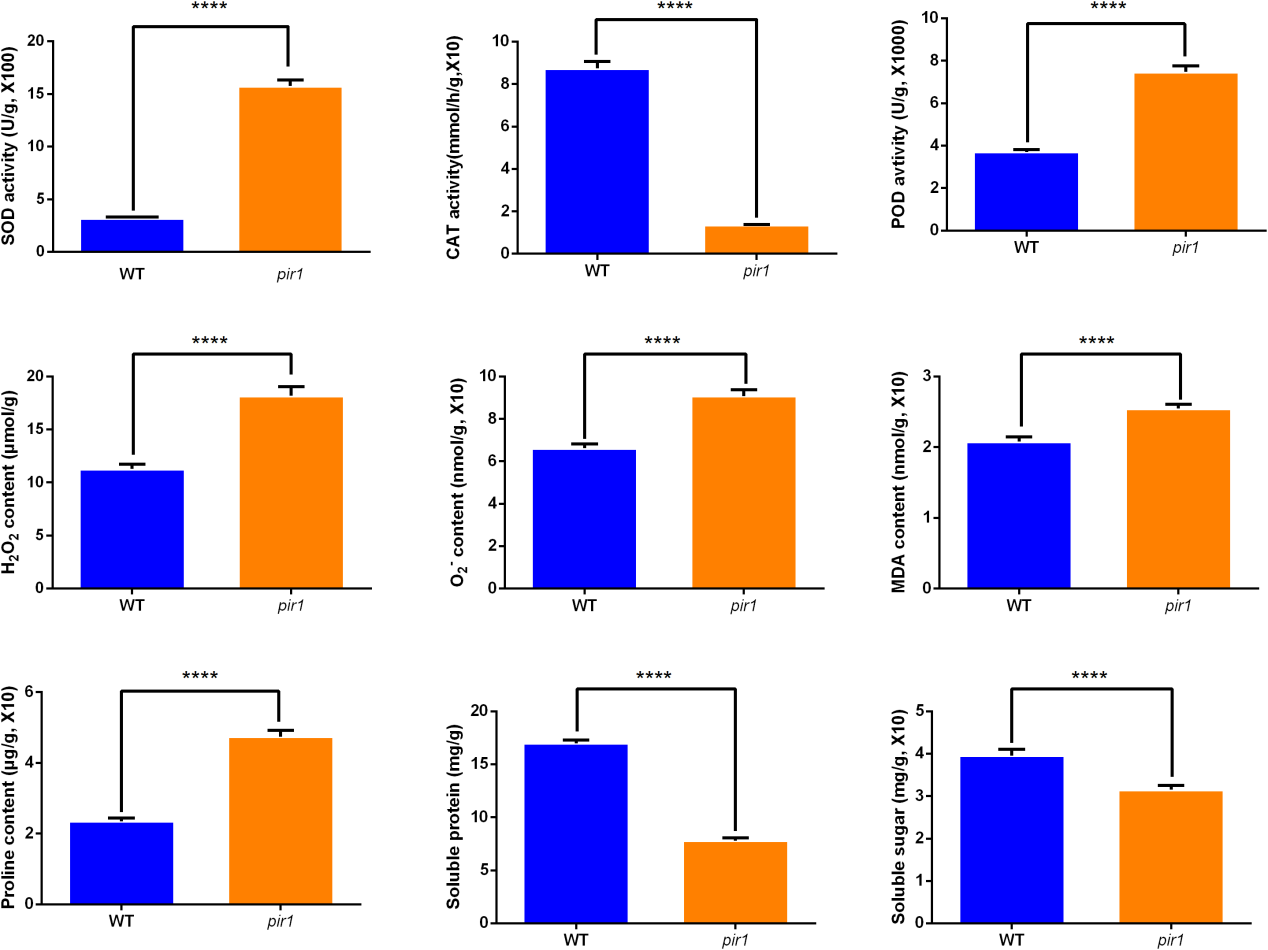
The activities of SOD, CAT, POD, and the levels of H_2_O_2_, O_2_·^−^, MDA, proline, soluble protein, soluble sugar in wild-type and mutant plants. ****p < 0.01.

As shown in **Figure 5**, the pir1 mutant exhibited significantly elevated levels of H_2_O_2_, O_2_·^−^ (superoxide anion), MDA, and proline compared to the wild type. This accumulation of ROS appears to trigger PCD, thereby playing a positive regulatory role in disease resistance. Both SOD and POD activities were significantly increased in *pir1*. These findings are largely consistent with data obtained at the transcriptional and translational levels.

SOD catalyzes the dismutation of O_2_·^−^ to H_2_O_2_. Consequently, the observed increase in SOD activity likely contributes to the higher H_2_O_2_ accumulation detected in pir1. In contrast, CAT activity was markedly and significantly reduced in the mutant compared to the wild type. This indicates a pronounced impairment in the mutant’s ability to scavenge H_2_O_2_, further exacerbating ROS accumulation.

Additionally, soluble protein and soluble sugar contents were higher in the wild type than in the mutant *pir1*. This suggests that the mutant experiences ROS-induced stress, potentially resulting in insufficient carbohydrate catabolism. This deficit may impair energy metabolism essential for vital cellular processes, contributing to the observed mutant phenotypes of dwarfism and sterility.

### 3.6 Analysis of photosynthetic indexes

**Figure 6:**
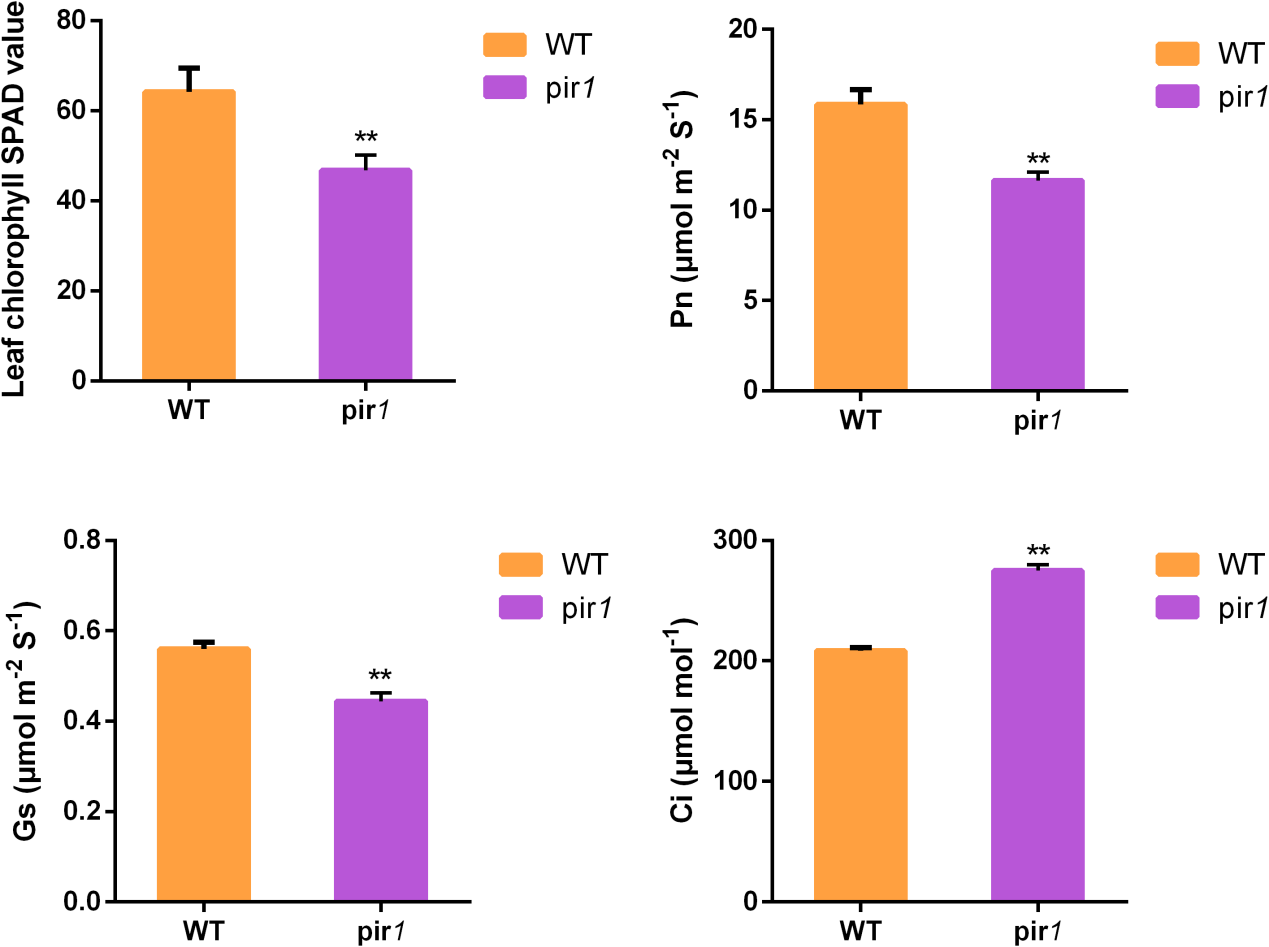
Assessment of chlorophyll SPAD values and photosynthetic parameters. **p < 0.01.

Proteomic analysis revealed the down-regulation of several key photosynthetic electron transport proteins, specifically PsbO, PsbP, PsaE and LFNR, in both leaf b and leaf c of the mutant *pir1*. Concurrently, multiple other proteins associated with photosynthesis were also down-regulated (**Supplementary Table S2**). Consequently, we measured SPAD values and photosynthetic parameters in both WT plants and the mutant *pir1*. The results demonstrated that SPAD values were significantly lower in the mutant *pir1* compared to the WT (**Figure 6a**). Similarly, Pn and Gs were also significantly reduced in *pir1* (**Figures 6b, c**). In contrast, Ci was higher in the WT than in the mutant (**Figure 6d**). These findings collectively indicate that photosynthetic pathways are substantially impaired in the mutant *pir1*, resulting in a severe reduction in photosynthetic capacity.

### 3.7 Observation of TEM

**Figure 7:**
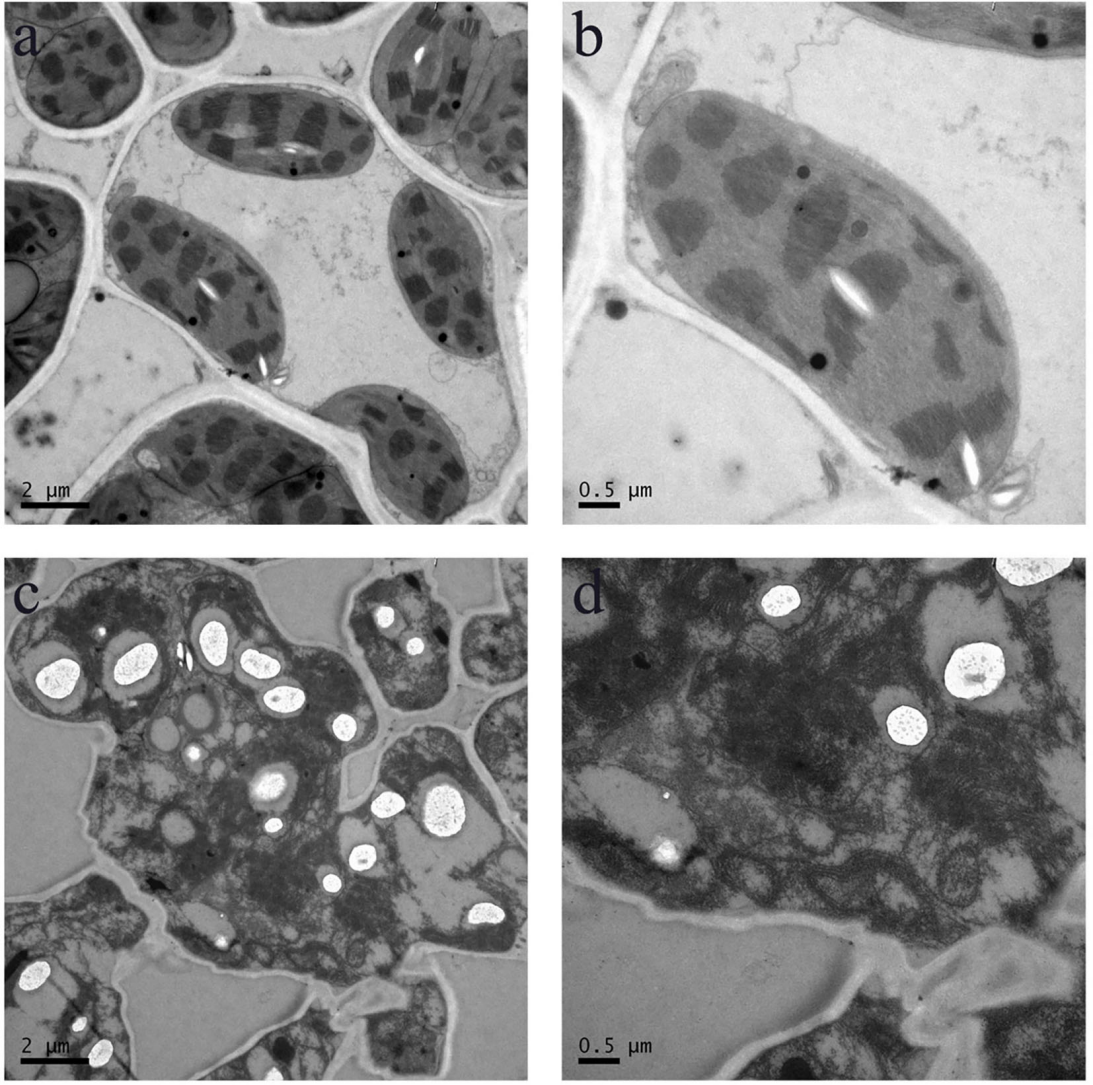
Cellular structure in the WT and *pir1*. **(a, b)** Cellular structure of central portion of WT. **(c, d)** Cellular structure of central portion of the *pirl*.

At the tillering stage, transverse sections were taken from the mid-section of leaves of both wild-type and mutant plants, and cellular structures were examined using TEM (**Figure 7**). The results revealed that in the wild-type, the cytoplasm was uniformly distributed, the grana lamellae of chloroplasts were well-organized, and the overall cellular architecture was intact. In contrast, the cellular structure of the mutant *pir1* was severely compromised. Cells exhibited significant shrinkage and underwent intense plasmolysis. Although the cell wall remained relatively intact, it displayed localized thickening and a loose structure. The plasma membrane detached from the cell wall, exhibiting invagination and vesiculation. The cytoplasmic matrix was thin and watery, containing numerous irregular vacuoles. The chloroplast envelope was largely ruptured, the thylakoid system was completely disintegrated into fragments, the stroma was vacuolated, and the grana thylakoids were disorganized and fragmented. Mitochondrial cristae appeared blurred and reduced. The central vacuole was reduced in volume, with its membrane structure appearing wrinkled.

## 4 Discussion

**Figure 8:**
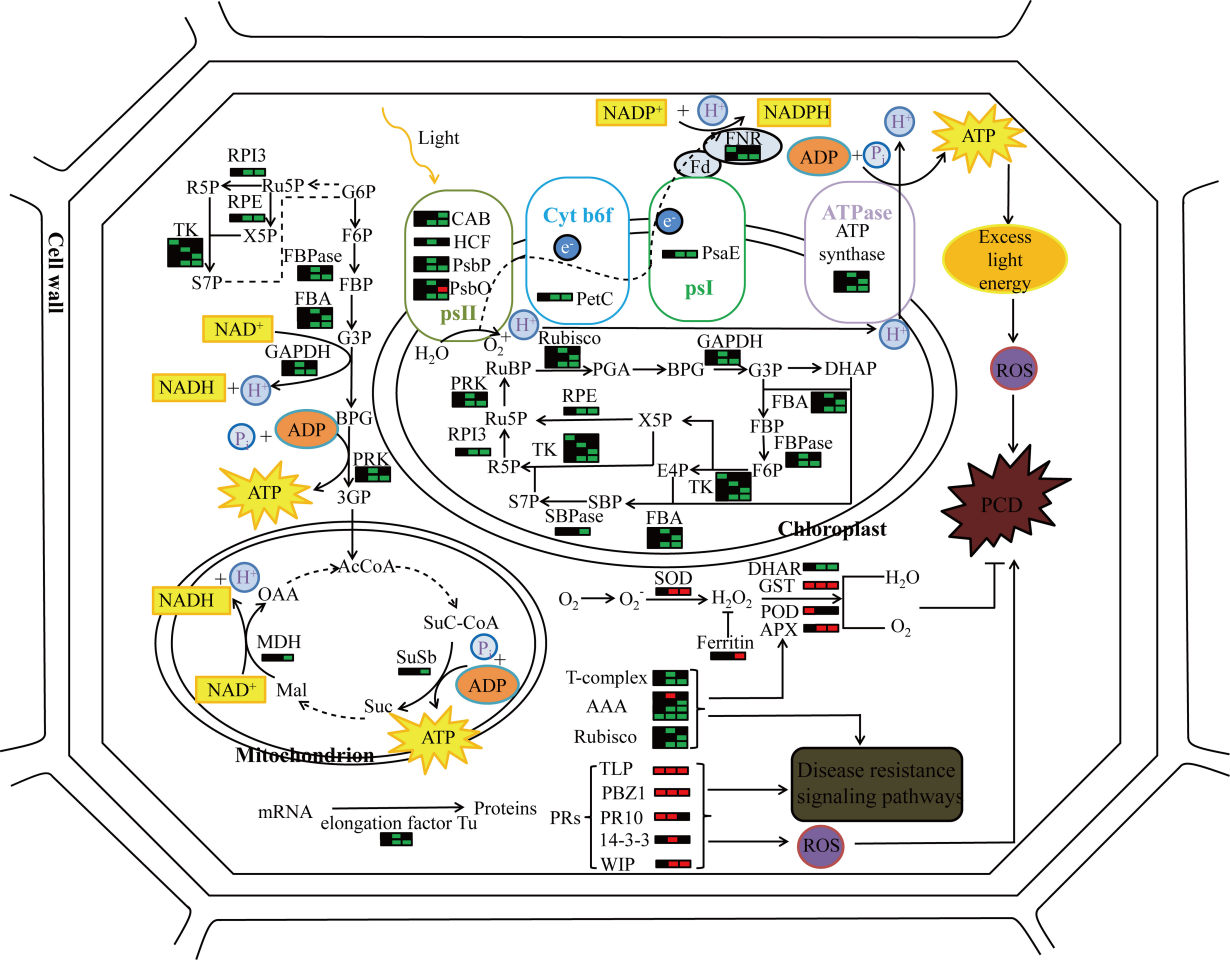
The molecular pattern of *pir1* triggering PCD at the protein level. Each bar graph represents a protein, the left side represents the flag leaf, the middle represents the 2nd leaf, the right represents the 3rd leaf, red represents up-regulation, green represents down-regulation, and black represents no change.

In proteomics studies of the mutant *pir1*, downregulation of numerous proteins related to photosynthesis and carbohydrate metabolism, alongside upregulation of defense-related proteins, was observed. These changes correlate with PCD phenotype observed in *pir1*. Based on these observations and existing literature, we propose a potential functional model (**Figure 8**). Downregulation of photosynthetic proteins may lead to reduced photosynthetic efficiency. Unutilized light energy could potentially promote the accumulation of ROS, a key signal triggering PCD. Concurrently, the upregulation of defense-related proteins may participate in enhancing disease resistance responses. These proteins themselves might generate ROS or participate in ROS signaling, thereby further regulating the progression of PCD. The upregulation of some redox-related proteins may help mitigate ROS accumulation, limiting the spread of lesions and thus offering some protection to the plant. The ensuing PCD process likely impacts carbohydrate metabolism in the mutant *pir1*, potentially causing significant disruption to cellular metabolism, including carbohydrate metabolism and energy production. It is hypothesized that this may prioritize the allocation of energy and carbon resources towards defense responses.

### 4.1 Photosynthetic related proteins

Research indicates that under strong light conditions, the closure of stomata in plant leaves leads to insufficient CO_2_ supply, resulting in reduced carbon assimilation efficiency (Keyse, 1992). The excess light energy causes a depletion of the electron acceptor NADP^+^. This deficiency forces Photosystem I (PSI) to transfer electrons to molecular oxygen (O_2_), reducing it to the superoxide radical (O_2_·^−^) (Mehta et al., 1992). Concurrently, excitation energy transfers the excess energy to ground-state oxygen, converting it into highly reactive singlet oxygen (¹O_2_). The O_2_·^−^ can react with compounds such as plastocyanin or cytochromes, generating more ROS molecules like H_2_O_2_ (Henkes, 2001; Scandalios, 1993). These ROS intensify peroxidation processes and further attack other cellular components in the plant, damaging protein structures and ultimately causing cellular injury (Kang et al., 2007).

In the mutant *pir1*, downregulation of multiple photosynthesis-related proteins was detected, including Ferredoxin-NADP^+^ Reductase (FNR), the large subunit of Ribulose-1,5-bisphosphate Carboxylase/Oxygenase (Rubisco-L), components of the Photosystem II Oxygen-Evolving Complex (OEC) and Oxygen-Evolving Enhancer (OEE) proteins, as well as Chlorophyll A-B Binding Proteins (CAB). This downregulation correlates with the PCD phenotype, suggesting impaired photosynthetic capacity in *pir1*. Considering the role of ROS in PCD, we speculate that the downregulation of photosynthetic proteins may reduce light capture and utilization efficiency, potentially increasing the risk of excess oxygen radical generation during photosynthetic electron transport. These accumulated ROS could be a key factor triggering PCD in *pir1* and simultaneously contributing to the observed enhanced disease resistance. However, it is equally reasonable that the occurrence of PCD may also lead to the down-regulation of these photosynthetic proteins. However, it is equally plausible that the occurrence of PCD causes the downregulation of these photosynthetic proteins. Validating this requires direct functional assays to establish causality for the identified proteins.

The mutant *pir1* exhibits phenotypes such as reduced photosynthetic efficiency and decreased chlorophyll content, indicating that *pir1* is essential for maintaining normal photosynthetic function. This suggests that *pir1* may influence the photosynthetic process through some mechanism. However, the specific molecular mechanisms by which *pir1* affects or participates in the photosynthetic pathway, such as its direct targets or regulatory modifications, will be a major focus for future research.

### 4.2 Defense related protein

Compared to the wild type, *pir1* exhibits differential abundance of several defense-related proteins, including: PRs, 14-3–3 proteins, thaumatin-like protein (TLP), wound-induced protein (WIP), T-complex protein, and AAA-type ATPase.

TLP is considered a member of the pathogenesis-related protein family 5 (PR-5), involved in defense responses and exhibiting antifungal activity against numerous plant pathogens. Studies report that the *TaTLP1* gene isolated from wheat participates in wheat’s defense response against leaf rust (*Puccinia triticina*). Furthermore, through the interaction of *TaTLP1* with *TaPR1*, which triggers ROS accumulation, *TaTLP1* positively regulates wheat resistance to leaf rust (Wang et al., 2020). Haru Kirino et al. found that three TLP proteins secreted by the pinewood nematode (*Bursaphelenchus xylophilus*) significantly induced cell death in tobacco leaves (Kirino et al., 2020).

14-3–3 proteins constitute a class of important phospho-serine/phospho-threonine binding molecules that play crucial roles in regulating plant development and defense response (Oh, 2010). Chang-Sik Oh et al. discovered that during the incompatible interaction between tomato (*Solanum lycopersicum*) and the fungal pathogen *Verticillium dahliae* carrying the *Avr9* effector, three 14-3–3 genes (*TFT1*, *TFT4*, and *TFT6*) were induced (Oh and Martin, 2011; Roberts and Bowles, 1999). These genes may play a role in regulating HR.

Therefore, based on the known functions of these proteins in defense and cell death, we speculate that the increased expression of these defense-related proteins in the mutant *pir1* may play a role in its enhanced defense response and could be involved in inducing or regulating the observed PCD process, potentially correlating with the increased resistance exhibited by *pir1*.

Notably, although R genes play crucial roles in disease resistance pathways, our proteomic analysis did not detect significant differential expression between wild-type and mutant plants. This observation could be attributed to several factors: Inherently Low Abundance: R proteins are typically expressed at very low levels. Even upon induction, their absolute abundance might fall below the reliable detection limit of mass spectrometry.Mechanism Independent of Abundance Changes: The core activation mechanism of R proteins primarily relies on post-translational modifications (PTMs) and conformational changes, rather than large-scale changes in total protein abundance. The key event is their activation triggered by effector recognition, leading to conformational shifts and PTMs. Consequently, the total protein levels may not exhibit significant upregulation within short timeframes. Function as Signaling Hubs: Activated R proteins function primarily as molecular switches and signaling platforms that recruit downstream components. “Sentry” Mode: Many R proteins are constitutively expressed and act as pre-formed “sentinels” surveilling the intracellular environment. Following activation, their primary responses involve modifications and oligomerization, rather than a significant increase. These hypotheses require further validation in future studies.

### 4.3 Redox-related proteins

SOD, an antioxidant enzyme, catalyzes the dismutation of the superoxide anion radical into oxygen and H_2_O_2_. Subsequently, enzymes including APX, glutathione transferase (GST), POD, and dehydroascorbate reductase (DHAR) can decompose H_2_O_2_ into water and oxygen (Hiraga et al., 2000). Upregulation of SOD was observed in the *pir1* mutant, which may promote the conversion of O_2_^−^ to H_2_O_2_ (**Figure 5**). Simultaneously, the upregulation of GST, POD, and APX suggests that *pir1* likely activated its antioxidant defense system. This may help scavenge excess H_2_O_2_ and other ROS, alleviating oxidative damage. This activation of the antioxidant system could be induced by PCD or photosynthetic imbalance, thereby offering some protection to the plant against death from severe oxidative stress.

### 4.4 Proteins related to energy metabolism

ATP synthase synthesizes ATP from Pi and ADP using the transmembrane proton gradient generated by photosynthesis (Boyer, 2000; Weber and Senior, 2000). In the mutant *pir1*, the downregulation of multiple ATP synthases results in insufficient energy supply within the plant. It is hypothesized that this energy deficiency contributes to the dwarfism of the mutant plants. The downregulation of multiple ATP synthase subunits indicates potentially limited ATP synthesis capacity in *pir1*.

ABC transporters are a class of ATP-driven pumps composed of two transmembrane domains and two ATP-binding domains (Jasinski et al., 2003). By binding and hydrolyzing ATP to release energy, ABC transporters translocate substrates-such as peptides, amino acids, sugars, alkaloids, glutathione, vitamins, and cellular metabolites-across membranes. This process facilitates critical physiological functions in plants, including the maintenance of cellular osmotic homeostasis and antigen presentation (Banasiak et al., 2020; Rinaldo et al., 2017; Verrier et al., 2008; Zhang et al., 2013). The ClpP protease is an ATP-dependent proteolytic enzyme that typically forms a proteolytic complex with AAA+ proteins. Members of the AAA+ protein family utilize energy derived from ATP hydrolysis to translocate protein substrates into the ClpP protease for degradation. This mechanism is essential for maintaining cellular homeostasis and energy regulation (Lozano-Durán and Robatzek, 2015; Xia et al., 2013). Sucher et al. demonstrated that introducing the wheat *Lr34* gene into maize conferred enhanced resistance to northern leaf blight. Similarly, *Lr34* expression in barley conferred resistance to leaf rust and powdery mildew. Krattinger et al. further identified abscisic acid (ABA) as a substrate for the *Lr34* transporter. *Lr34*-expressing rice lines exhibited increased ABA accumulation, indicating *Lr34* mediates ABA redistribution to enhance biotic stress tolerance. Silencing genes encoding ABCG/PDR-type transporters increases disease susceptibility (Boni et al., 2018; Krattinger and Kang, 2019; Sucher et al., 2017). For example, silencing both *NaPDR1* and *NaPDR1-like* in wild tobacco following *Alternaria alternata* infection compromised resistance, impaired growth, and increased foliar disease severity, confirming PDR transporters function in biotic stress responses (Xu et al., 2018).

Therefore, we speculate that the downregulation of ABC transporters and ClpP protease disrupts cellular homeostasis and energy balance, potentially triggering cell death within the plant. This may contribute to the induction of PCD and the dwarfism and sterility phenotypes of the mutant. The downregulation of ABC transporters and ClpP protease likely interferes with intracellular homeostasis maintenance and ATP-dependent metabolic processes. Collectively, the downregulation of these energy metabolism-related proteins may lead to insufficient cellular energy supply and metabolic dysregulation in *pir1*. This state of energy and metabolic imbalance, along with potentially compromised protein homeostasis, could be a factor promoting PCD initiation. However, while potential energy limitation might be one factor contributing to dwarfism and poor fertility, other mechanisms such as hormonal imbalance or cell death in meristematic/reproductive tissues could equally play significant roles.

### 4.5 Proteins related to carbohydrate metabolism

The primary physiological function of carbohydrates is to provide energy required for biological activities. Glycolysis is an essential stage for glucose catabolism in all organisms. In strawberry plants infected with *C. fragariae*, most glycolysis-related enzymes showed a significant decrease by 72 hours post-inoculation (HPI). However, aldolase (ALD) and phosphoglycerate kinase (PGK) exhibited a transient increase at 24 and 48 HPI before declining, while alcohol dehydrogenase (ADH) progressively declined throughout the infection period. This downregulation of ADH may enhance plant defense responses, suggesting that glycolytic inhibition could limit the pathogen’s energy supply and thereby promote its fungal glycolysis (Fang et al., 2012; Pathuri et al., 2011). In the mutant *pir1*, proteins involved in this pathway, such as phosphoglycerate kinase (PGK), transketolase (TK), fructose-1, 6-bisphosphate aldolase (FBA), and phosphoribulokinase (PRK), were down-regulated. This indicates that carbohydrate metabolism may be broadly suppressed. Metabolic suppression could further exacerbate the energy supply shortage.

The pentose phosphate pathway (PPP) represents an alternative route for glucose oxidation, providing reducing power for various cellular reactions and maintaining the reduced state of glutathione to prevent membrane lipid peroxidation (Fang et al., 2012). Therefore, it is hypothesized that the downregulation of PPP enzymes, such as ribulose-5-phosphate 3-epimerase (RPE), in *pir1* suppresses this pathway. This could disrupt the glutathione redox balance, potentially causing membrane lipid peroxidation, insufficient carbohydrate breakdown, and energy deficiency. We hypothesize that this may contribute to phenotypic abnormalities.

The Tricarboxylic Acid (TCA) cycle is the final metabolic pathway for carbohydrates, lipids, and amino acids, and serves as the central hub connecting these three metabolic streams. Within this cycle, downregulation of enzymes like Malate dehydrogenase (MDH) and Succinyl-CoA synthetase (SuSc) also reduces the production of energy required for vital activities and may contribute to the dwarfism and sterility of the mutant plant. However, it is worth noting that these phenotypes could also stem directly from hormonal changes or cell death events in meristematic or reproductive tissues. Determining the primary causative factor for dwarfism, sterility, and other phenotypes requires further experimental evidence.

## 5 Conclusions

Our study demonstrates that the mutant *pir1* exhibits strong resistance to *Xoo*. Proteomic analysis revealed downregulation of proteins associated with photosynthesis, carbohydrate metabolism, and energy metabolism in *pir1*, alongside upregulation of defense-related proteins. Concurrently, key photosynthetic parameters were significantly reduced compared to the wild type, indicating diminished photosynthetic efficiency. This impairment in light energy utilization likely leads to excess excitation energy, triggering ROS accumulation and potentially inducing PCD. Furthermore, the upregulation of defense-related proteins in pir1 may contribute to enhanced apoptosis, ultimately resulting in significantly improved resistance to bacterial blight.

## Data Availability

The original contributions presented in the study are included in the article/**Supplementary Material**. Further inquiries can be directed to the corresponding authors.
