# Supplementary material for: Proteomic analysis of rice mutant pir1 reveals molecular mechanisms triggering PCD and conferring high resistance to bacterial blight
Source: Front Plant Sci. 2025 Aug 28;16:1652068. doi: 10.3389/fpls.2025.1652068 (PMC12424556; doi:10.3389/fpls.2025.1652068)
Supplement: Supplementary file 1 [file DataSheet1.docx]

Supplementary Material

# Supplementary Data

Supplementary Material should be uploaded separately on submission. Please include any supplementary data, figures and/or tables.

Supplementary material is not typeset so please ensure that all information is clearly presented, the appropriate caption is included in the file and not in the manuscript, and that the style conforms to the rest of the article.

# Supplementary Figures and Tables

For more information on Supplementary Material and for details on the different file types accepted, please see [here](https://www.frontiersin.org/guidelines/author-guidelines#supplementary-material).

## Supplementary Figures

**
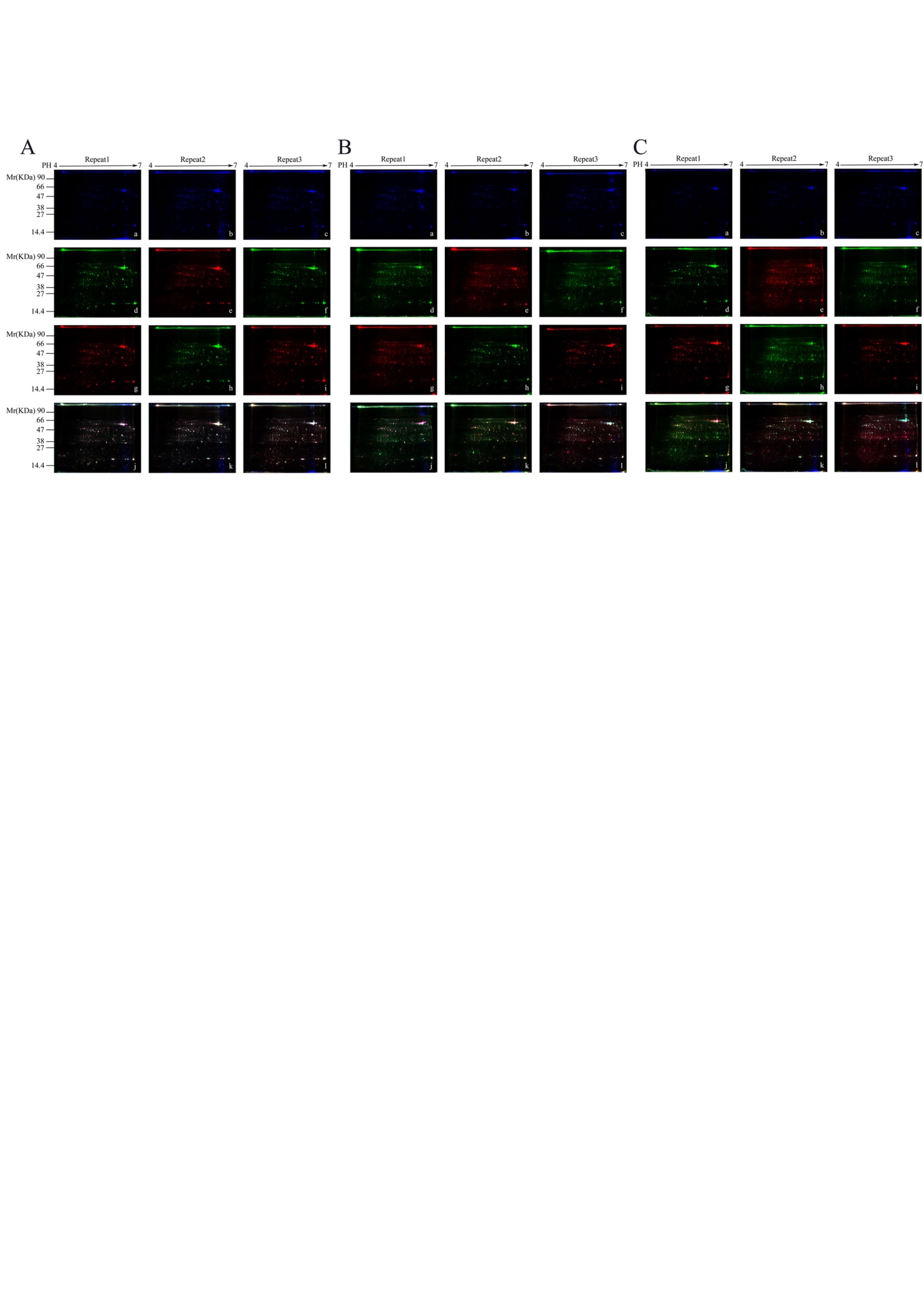
**

**Supplementary Figure 1.** 2D-DIGE images of leaf position a (A), b (B), and c (C). Blue was Cy2 labeled three groups of repeated equal amounts of mixed protein, green was Cy3 labeled protein, and red was Cy5 labeled protein. a, b, c, were three sets of gel images of repeated equal amounts of mixed protein. d, e, f were three repeated gel images of ZJ22 leaf protein. g, h, i were three repeated gel images of *pir1* leaf protein. j, k, l, were three overlapping gel images of three fluorescent label


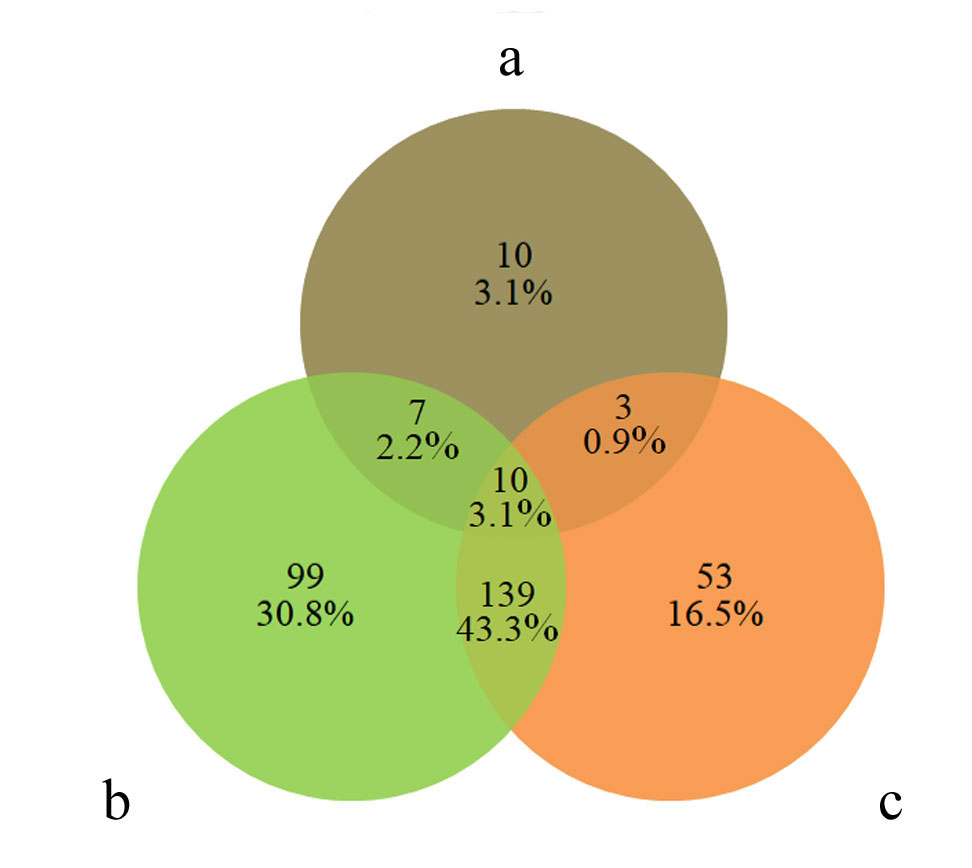


**Supplementary Figure 2.** Differentially expressed proteins in different leaf positions. (a) was the differential protein on the flag leaf; (b) was the differential protein on the 2nd leaf; and (c) was the differential protein on the 3rd leaf.

**Table S1.** Primers are listed for real-time PCR.

| Gene ID | Description | Primer sequence (From 5′ to 3′) |
| --- | --- | --- |
| Os09g0467200 | Tau class glutathione S-transferase 17 | F: CTGAAGAGAAGTGCAACGCTG  R: CGAGAGGATCTGCTCACGG |
| Os07g0694700 | Ascorbate peroxidase | F: AACTTCCCATCCTCTCCTAC  R: CAAAGAAGGCGTCCTCATC |
| Os05g0323900 | Manganese-superoxide dismutase | F: AGGTGCTGCTTTACAAGGA  R: CCCAACAAAGGAACCAAGTT |
| Os03g0786100 | Glycolate oxidase | F: GAACATACGGCCGGCGAAGA  R: GGATTTGCTTGAGCACGGCG |
| Os01g0106400 | Isoflavone reductase-like gene | F: TCGCCTCAACTGGACCAGGA  R: TGAACCCAGCTCTCTCGGCT |
| Os10g0355800 | ATP synthase subunit beta, putative | F: GCTCCTTATCGGCGTGGAGG  R: ATACGGATACGCCCCCGTGA |
| Os07g0492000 | Nucleoside diphosphate kinase 1 | F: CCTCATCGCCTCCGCTTCTG  R: GGTCGGCATAGTGCTGCTGT |
| Os02g0595700 | Chloroplast elongation factor | F: GGCCAACCCTGCCATTAAGC  R: AGCAAGCAAGAACGGGAGGT |
| Os07g0141400 | Photosystem II subunit PsbP | F: AGTTCCCCGGACAGGTCCTC  R: GGGAGCCGAACTCGGTGATG |
| Os01g0501800 | Oxygen-evolving complex | F: CCGTGGTTCGTCCTTCCTCG  R: CCAATCACCTCGCCGGTCTC |
| Os12g0555200 | Pathogenesis-related Bet v I family protein | F: GCTCACGGTGGAGTACGAGC  R: CGTACTCAGCAGGGTGAGCG |
| Os02g0580300 | G-box factor 14-3-3 homologs | F: GATATTGCCCTGGCAGAGTTG  R: GAGATATCGGAAGTCCACAGC |
| Os03g0710800 | G-box factor 14-3-3 homologs | F: AGCAGCTGAGAACACTCTTG  R: CAGCAATAGCATCGTCGAAC |
| Os11g0592000 | Pathogenesis-related (PR) protein 4 | F: TCGTGGCGTCAGAAGTATGG  R: AGCCTGACCCCTAGGACCAA |
| Os06g0114000 | Protein HEAT INTOLERANT 4 | F: AGGGTAAAAGCTGCCAAGGA  R: TTCCTTCCCTTTGGTCCCAG |
| Os02g0768600 | Inorganic pyrophosphatase | F: TGAGCTTGACTGGAAAATTGTG  R: GCTTCTCAACATCATCCACATC |
| Os07g0677200 | Peroxidase | F: GCCTCGCAGAACTTATCGG  R: CGTTGTAGATCCTGTCCCTGA |
| Os12g0106000 | Iron storage protein ferritin 2 | F: AAGTACCAGAACATGCGTGGAGGCA  R: TTCGAGAGCCAAGGCCAACTCCATA |
| Os08g0126300 | Glyceraldehyde-3-phosphate dehydrogenase C7 | F: GTCGTCCTCGCATCTCCA  R: CGCAAAGGCCACGTTATT |
|  | *Actin* | F:GAGTATGATGAGTCGGGGTCCAG  R: ACACCAACAATCCCAAACAGAG |
